# Lamina Reimplantation Using Synthes Cranial Plate Post Laminectomy in the Treatment of Intradural Spinal Tumours: A Case Series and Review of the Literature

**DOI:** 10.7759/cureus.88233

**Published:** 2025-07-18

**Authors:** Ray Ern Chung, Emma Toh, Su Lone Lim, Nivedh Dinesh, Arjun Bolem, Shiong Wen Low, Ira Sun, Chun Peng Goh

**Affiliations:** 1 Neurosurgery, Ng Teng Fong General Hospital, Singapore, SGP; 2 Neurosurgery, National University Hospital, Singapore, SGP

**Keywords:** lamina reimplantation, laminectomy, laminoplasty, spinal tumours, spine surgery

## Abstract

We present a case series of patients with intradural spinal tumours who underwent posterior laminectomy followed by lamina reimplantation. This technique demonstrated favourable post-operative MRI visualisation and minimal adhesions. The primary objective of this study was to evaluate the potential benefits of lamina reimplantation following laminectomy in the surgical treatment of intradural spinal tumours. This was compared to conventional laminectomy techniques without reimplantation. A retrospective review of five patient cases was conducted, examining clinical notes and radiological imaging. Additionally, a literature review was performed to contextualise the findings within existing knowledge. Five patients with intradural spinal tumours underwent posterior laminectomy with lamina reimplantation. Post-operative MRI imaging showed excellent clarity, with minimal artefacts, enabling effective monitoring for residual or recurrent tumour. One patient developed tumour recurrence, necessitating reoperation. Notably, during re-exploration, there were no adhesions between the dura and reimplanted bone, significantly facilitating surgical access. The literature supports these observations, suggesting that lamina reimplantation may reduce postoperative spinal instability and epidural fibrosis, and it avoids the imaging artefacts commonly associated with metallic instrumentation. Lamina reimplantation after laminectomy in patients with intradural spinal tumours may offer significant advantages, including preservation of spinal stability, reduced scar tissue formation, and improved post-operative imaging. Further comparative studies on a larger scale are needed to substantiate these findings and guide best surgical practices.

## Introduction

Posterior laminectomy represents the approach of choice in accessing intradural spinal tumours [1,2]. It was first described for use in the removal of an intradural myxoma in 1887 [3]. While intradural spinal cord tumours are usually benign in nature, their local compressive effects on surrounding structures can lead to complications such as myelopathy or radiculopathy, necessitating the need for surgery [4].

Despite its widespread use in clinical practice, existing literature describes potential complications of this approach. This is attributed to the disruption of the posterior spinal tension band post laminectomy, which can in turn lead to spinal instability [5], post-operative kyphosis [6], epidural fibrosis, and dural adhesions [7], which may even contribute to failed back surgery syndrome (FBSS) [8].

To circumvent these complications, laminoplasty with lamina reimplantation can be done. This was first described in 1976 by Raimondi et al. [9] and has been shown to reduce the occurrence of iatrogenic spinal stenosis and posterior soft tissue adhesions [10].

We present five cases of intradural spinal tumours, which were operated on via a posterior laminectomy approach, after which the resected laminae were reimplanted and secured with Synthes cranial plates and screws (DePuy Synthes, Johnson & Johnson, New Brunswick, NJ), and describe two cases in detail. The benefits and limitations of lamina reimplantation post laminectomy are discussed in comparison to a traditional laminectomy, durotomy and tumour resection without reimplantation.

## Case presentation

Five cases of patients with intradural spinal tumours who underwent lamina reimplantation post laminectomy were retrospectively reviewed (Table 1). Two of these cases are detailed in the following case summaries.

**Table 1:** Summary of five cases, with each patient’s demographic, presenting symptoms, histological diagnosis, and effect on post-operative imaging.

Case	Age	Sex	Presenting symptoms	Laminoplasty site	Diagnosis	Post-operative imaging
1	56	F	Incidental radiological finding of intracranial lesions, upper limb weakness, and hyperreflexia	Cervical 3 to cervical 5	C3/4 intradural schwannoma	MRI cervical spine done 1.5 months post-operatively – tumour recurrence noted, no artefacts
2	58	F	Falls, lower limb weakness, and numbness	Thoracic 11 to lumbar 1	T11/12 WHO grade 12 haemangioblastoma	MRI thoracic spine done post-operatively – reduction of tumour size, no artefacts
3	60	M	Myelopathic symptoms and hyperreflexia	Thoracic 7 to thoracic 8	T7/8 intradural schwannoma	MRI thoracic spine done on post-operative day 3 and 1 year post-operatively – no recurrence or artefact seen
4	52	F	Foot drop with subacute urinary and bowel incontinence	Cervical 5 to thoracic 3	C4 to T3 WHO grade 2 spinal ependymoma	MRI cervical spine done on post-operative day 1 – no artefact, laminoplasty sites well visualised
5	66	M	Thoracic myelopathy	Thoracic 1 to thoracic 3	T1-T3 WHO grade 2 spinal ependymoma	MRI thoracic spine at post-operative day 1 and 3-month follow-up – no artefacts, no recurrence

Case 1

**Figure 1:**
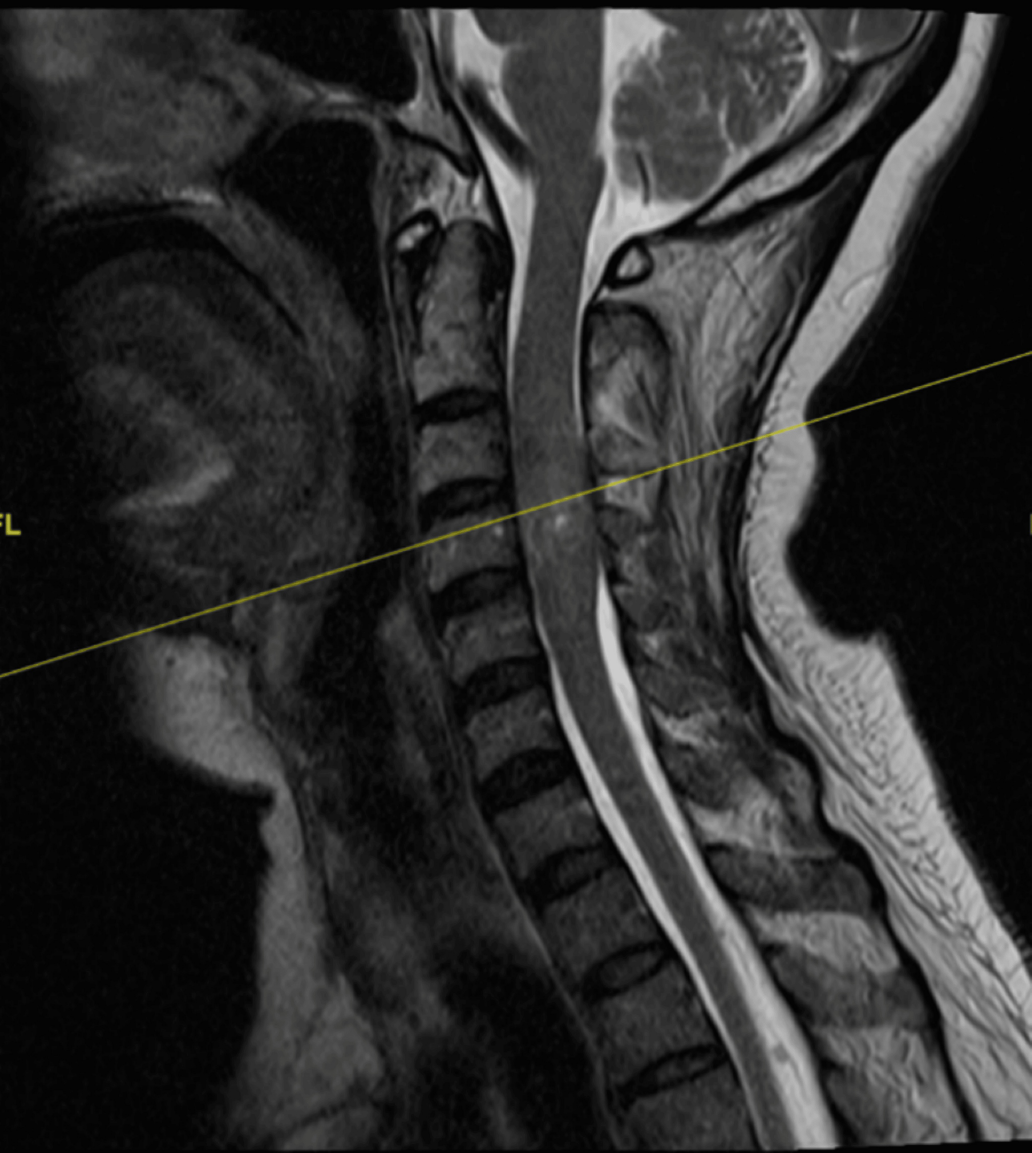
Pre-operative sagittal T2-weighted MRI of intradural extramedullary lesion at C3-4 level.

**Figure 2:**
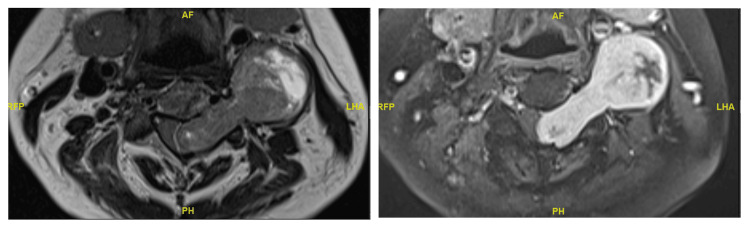
Pre-operative axial MRI images of intradural extramedullary lesion at C3-4 level.

A 56-year-old female patient presented with left-sided weakness. She was found to have multiple skin lumps over the right shoulder, right posterior thigh and posterolateral aspect of the right proximal leg, which were well-defined, not attached to underlying structures and had no overlying skin changes. She then underwent a CT of the neck, thorax, abdomen, and pelvis, which revealed multiple heterogeneously hypodense lesions in the anterior mediastinum, posterior mediastinum, and retroperitoneum. Further MRI of the brain and whole spine scans revealed multiple intracranial lesions in the right parafalcine region, anterior and left middle cranial fossae, as well as multiple well-defined intradural extramedullary mass lesions and extradural masses of varying sizes seen throughout the spine. Notably, the lesions at the C3-4 level were compressing the spinal cord and displacing towards the right side (see Figures 1, 2 for pre-operative MRI).

Initial neurological examination revealed hyperreflexia over the left side and a slight weakness over left elbow extension, which scored a 4+ out of 5 on the Medical Research Council (MRC) scale. Over the subsequent month, the patient experienced progressive neurological deterioration in her left upper limb, with muscle strength declining to MRC grade 4/5 across all C5 to T1 myotomes. Coordination of the left upper limb also worsened, and she was noted to exhibit bilateral hypertonia. Given her progressive neurological deterioration, she underwent debulking of the left C3/4 lesion.

**Figure 3:**
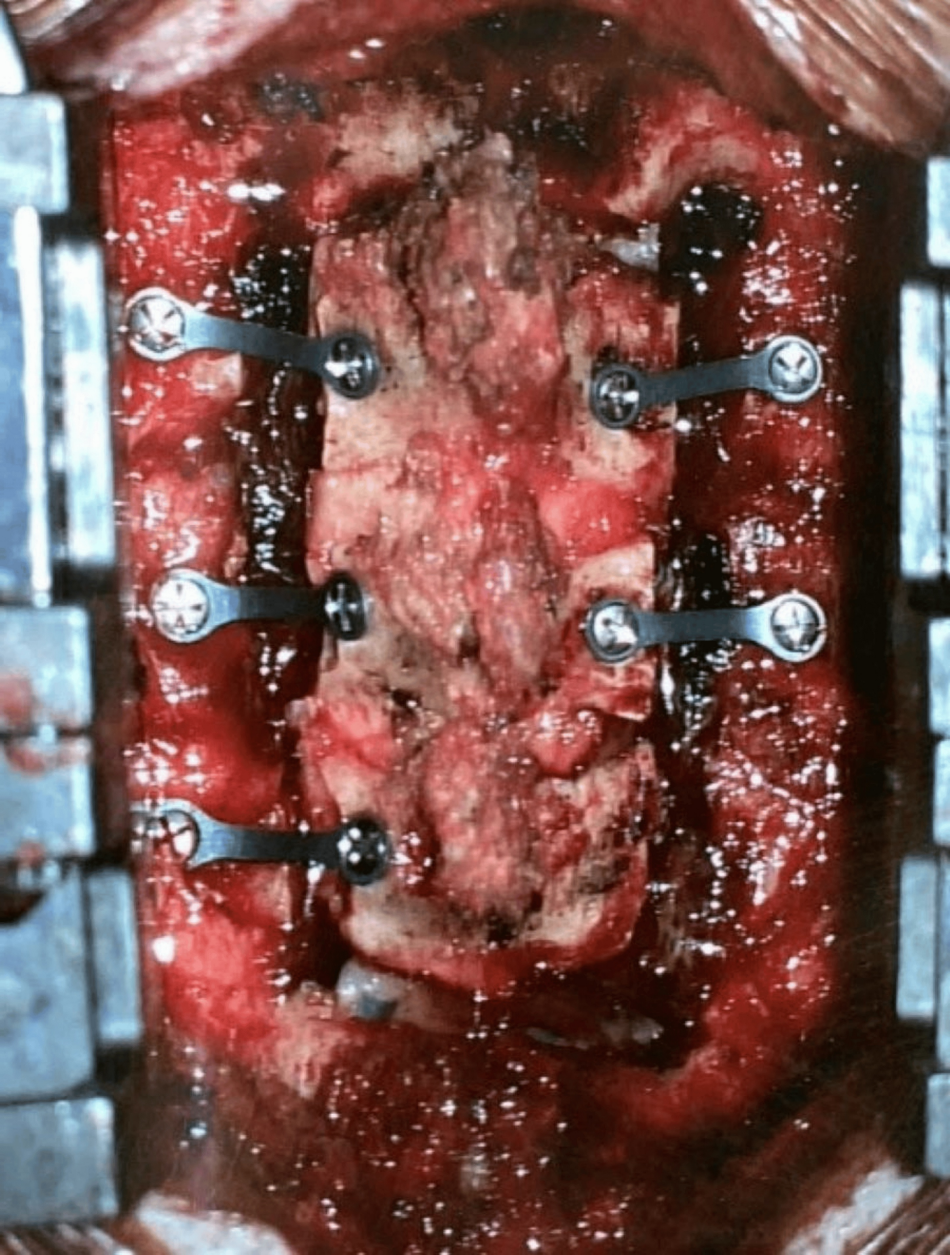
Replacement of C3-5 laminae post laminectomy, secured with Synthes cranial plates and screws.

Surgery was performed under general anaesthesia with the patient's head secured to the operating table using a radiolucent clamp. Intraoperative neuromonitoring was utilised throughout the procedure. A midline skin incision was made along the avascular plane of the ligamentum nuchae, followed by subperiosteal dissection to expose the cervical lateral masses. Bilateral troughs at the C3 to C5 laminae were created using a 2.2 mm match-head high-speed drill to facilitate their separation from the lateral masses. The C3 to C5 laminae were subsequently removed en bloc following careful detachment of the underlying ligamentum flavum. The underlying dura remained intact. After removal of the intracanal portion of the tumour, the dura was closed primarily in water-tight fashion, and the lamina was replaced and secured with Synthes low-profile cranial plates and screws (Figure 3). The histology of the tumour was schwannoma.

**Figure 4:**
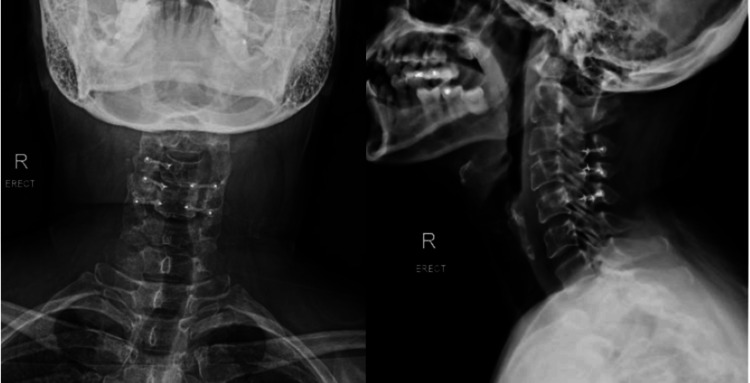
Post-operative X-ray images demonstrating satisfactory placement of Synthes cranial plates and screws.

**Figure 5:**
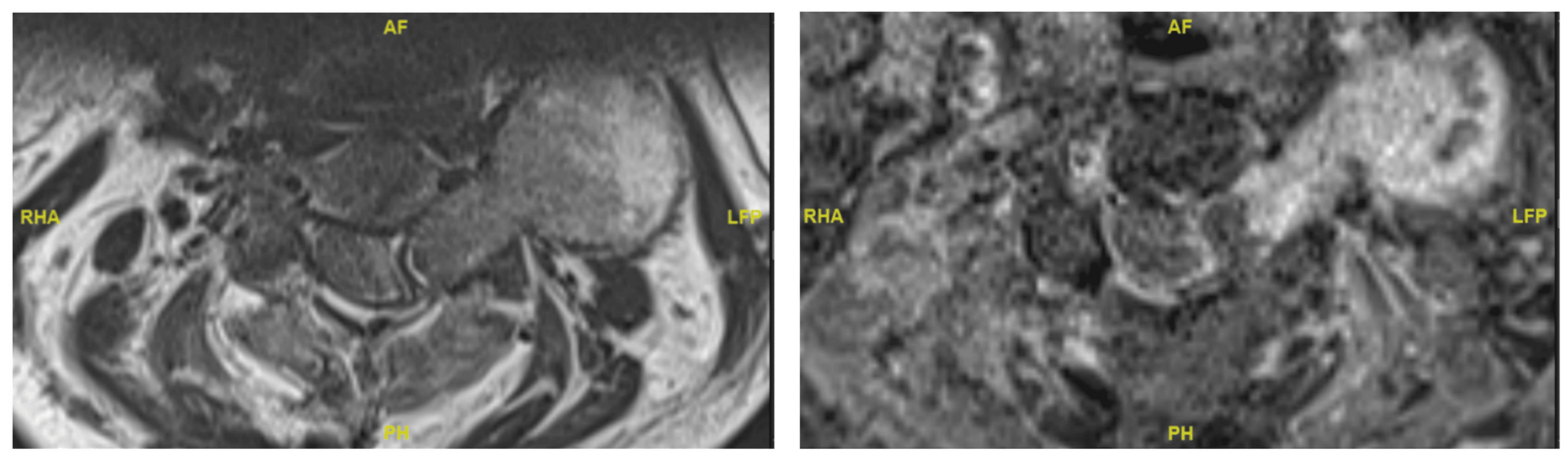
Post-operative axial MRI images of intradural extramedullary lesion at C3-4 levels showing interval increase in space available for cord and notable absence of metal artefacts.

**Figure 6:**
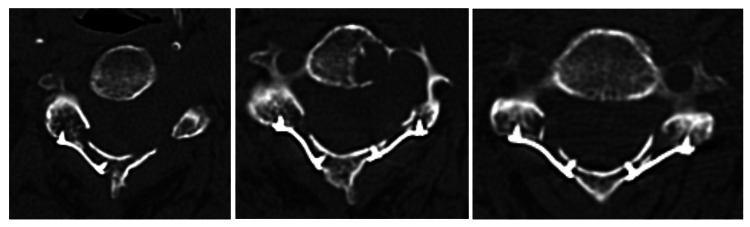
Post-operative CT images of screws at C3 (left), C4 (middle), and C5 (right) levels.

Post-operative X-ray images showed satisfactory placement of Synthes cranial plates and screws (see Figure 4 for post-operative X-ray). Post-operative MRI revealed an interval reduction in tumour size and an increase in space available for the cord. Notably, there was an absence of metal artefacts despite the use of Synthes plates and screws (see Figures 5, 6 for post-operative MRI). She was discharged on post-operative day seven.

**Figure 7:**
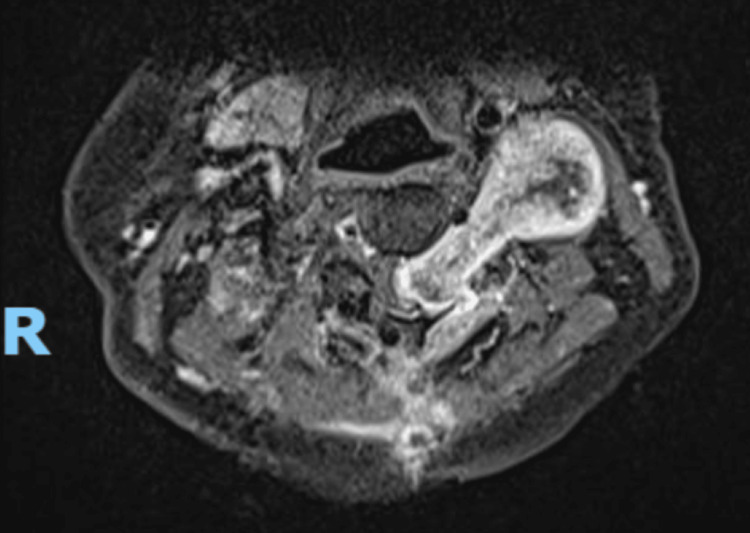
Pre-operative MRI prior to second surgery suggesting tumour recurrence.

Over the next 1.5 months, she experienced worsening left-sided upper limb weakness with neck and shoulder pain. Physical examination revealed MRC grade 2 for shoulder abduction and MRC grade 4 across C6 to T1 myotomes. Repeat MRI revealed recurrence of the tumour at the surgical cavity (see Figure 7 for pre-operative MRI), and she underwent a repeat debulking surgery. Intraoperatively, the authors observed an absence of adhesions between the bone and dura, which facilitated a smoother and less technically demanding surgical dissection. Due to the atypically rapid growth of the schwannoma, the patient underwent stereotactic radiotherapy targeting the residual tumour.

Case 2

**Figure 8:**
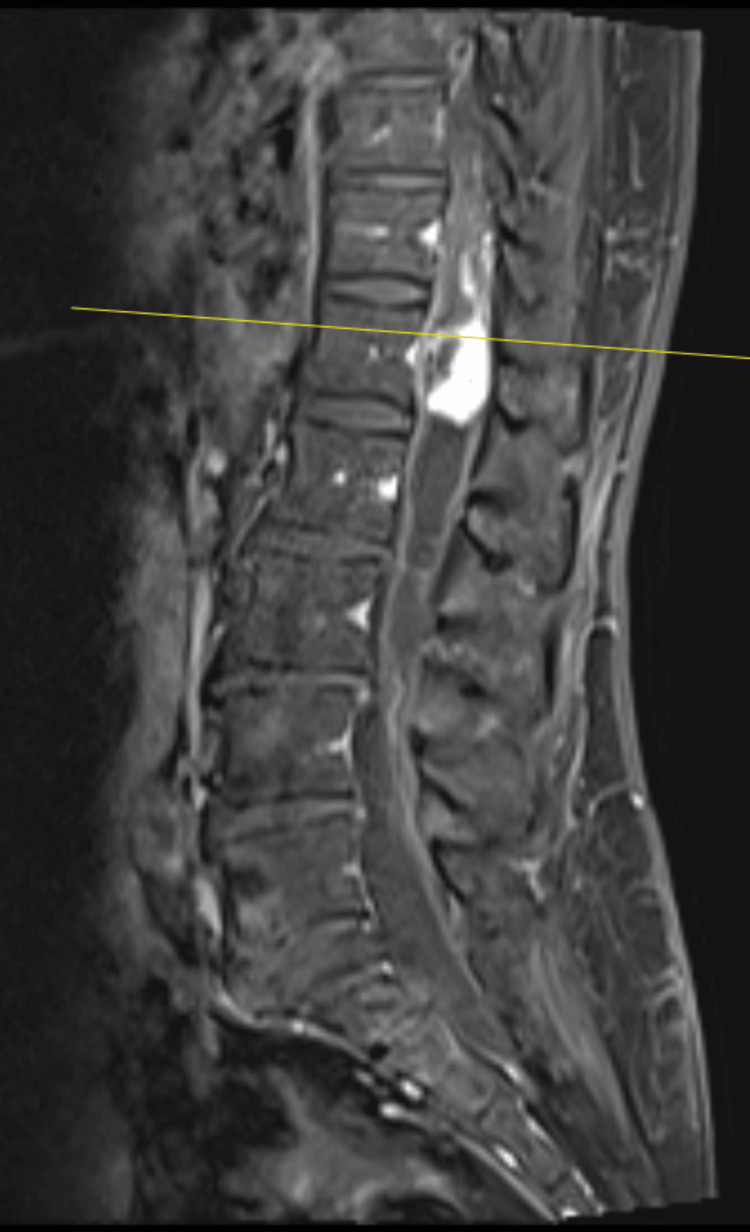
Pre-operative T2-weighted sagittal MRI image of intradural lesion at the level of T12.

**Figure 9:**
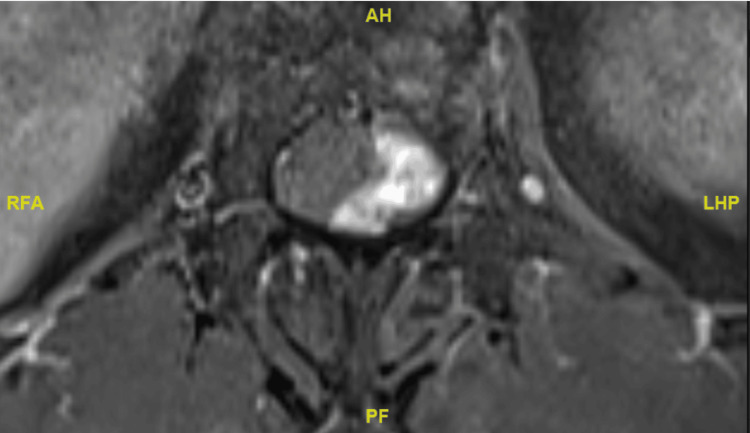
Pre-operative T1-weighted axial MRI image of intradural lesion at the level of T12.

**Figure 10:**
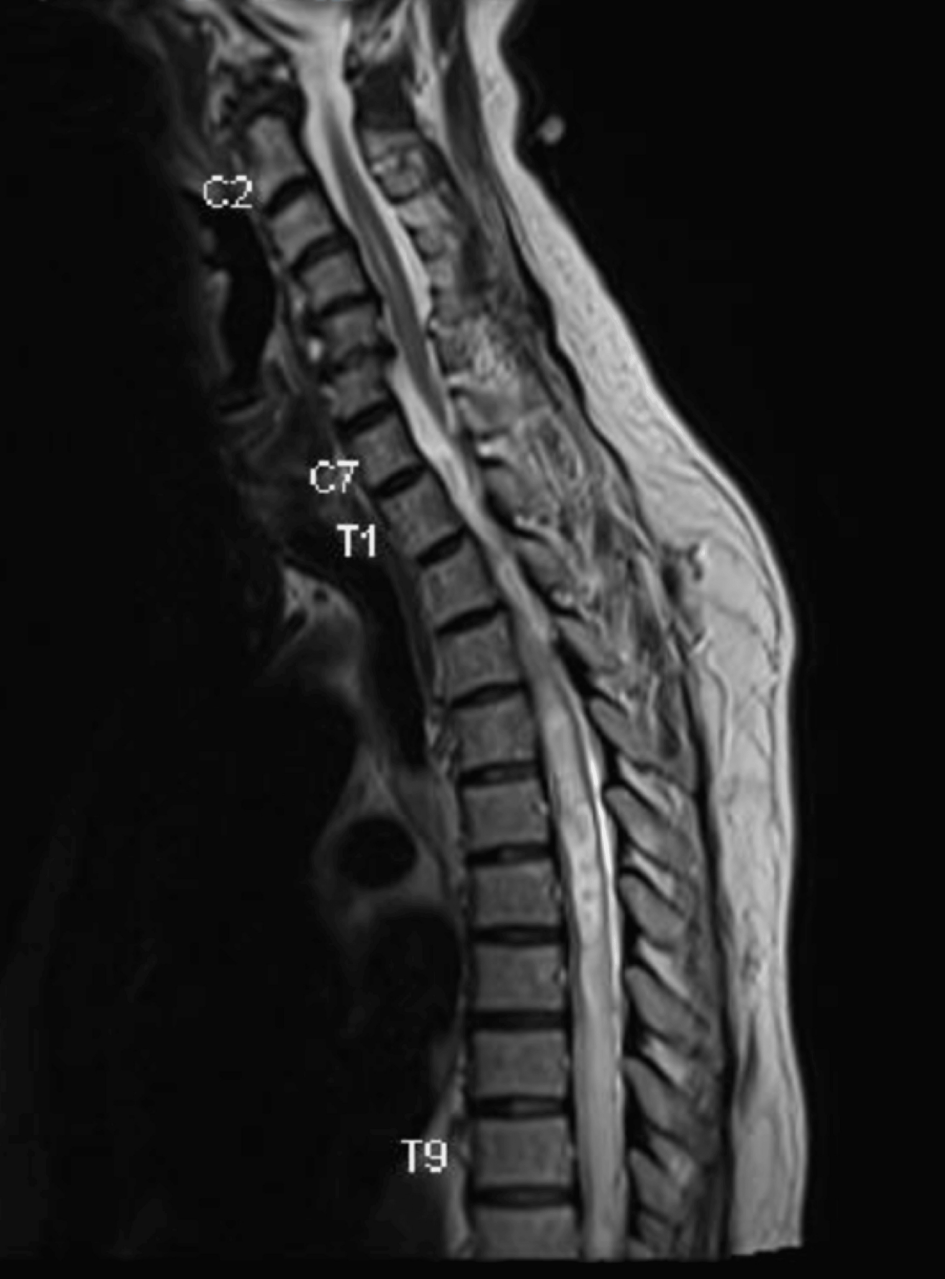
Pre-operative T2-weighted sagittal MRI image showing extensive syrinx.

A 58-year-old female patient presented with a two-year history of left lower limb paraesthesia and left foot drop. Neurological examination revealed a left foot drop, with ankle dorsiflexion and hallux extension graded 1/5. There was patchy sensory loss in the left lower limb, while anal tone and perianal sensation remained intact. An MRI of the spine showed a lobulated, heterogeneously enhancing solid-cystic lesion at the T11 and T12 level, which was associated with extensive syrinx (see Figures 8-10 for pre-operative MRI).

**Figure 11:**
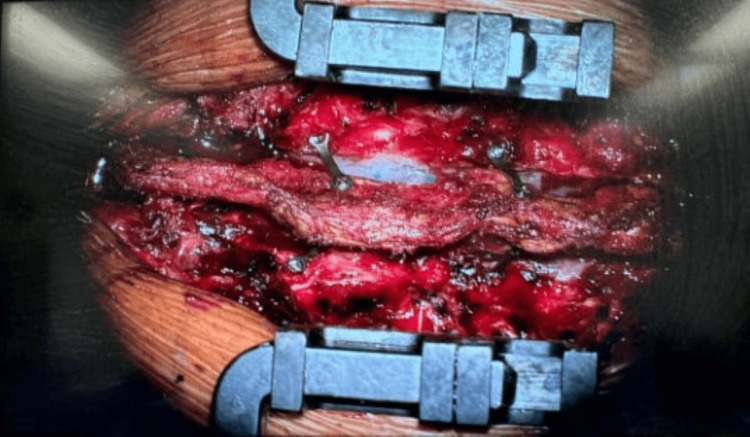
Post-operative photograph of replacement of T12 laminae post laminectomy, secured with Synthes cranial plates and screws.

Similar to the previous case, the T11 to L1 lamina was removed en bloc, followed by excision of the tumour. The lamina was subsequently replaced back in its original position and secured with Synthes cranial plates and screws (see Figure 11 for post-operative photograph). The histology of the tumour was consistent with a CNS WHO grade 1 haemangioblastoma.

**Figure 12:**
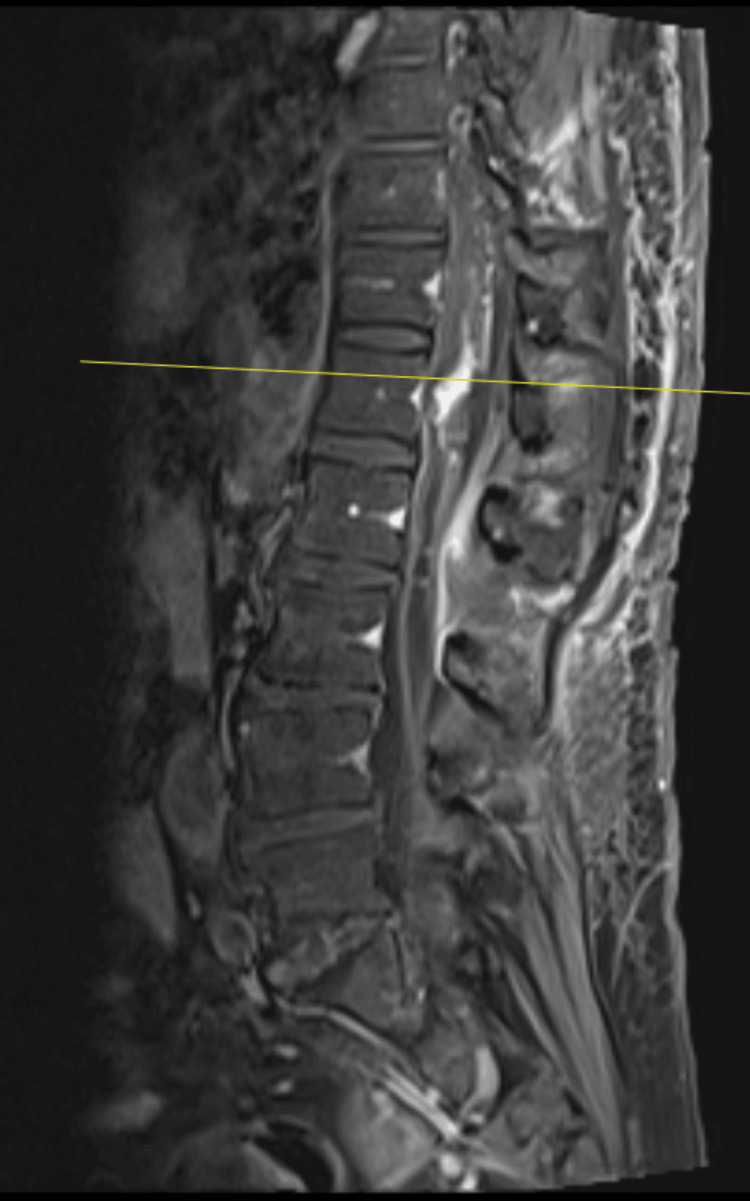
Post-operative sagittal MRI image of intradural lesion at the level of T12 with a notable absence of metal artefacts.

**Figure 13:**
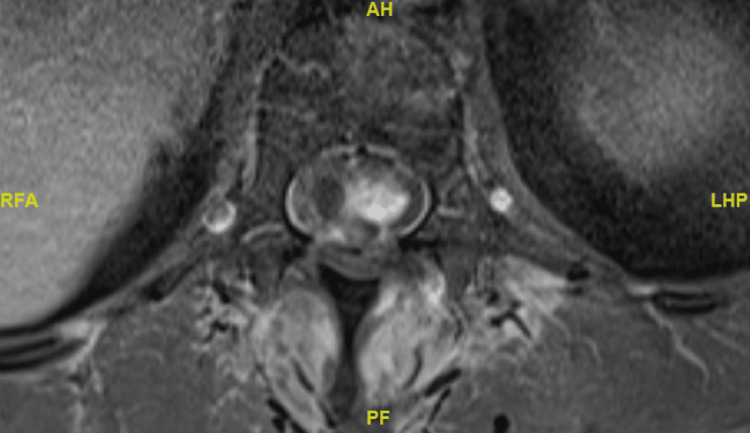
Post-operative axial MRI image of intradural lesion at the level of T12 with a notable absence of metal artefacts.

**Figure 14:**
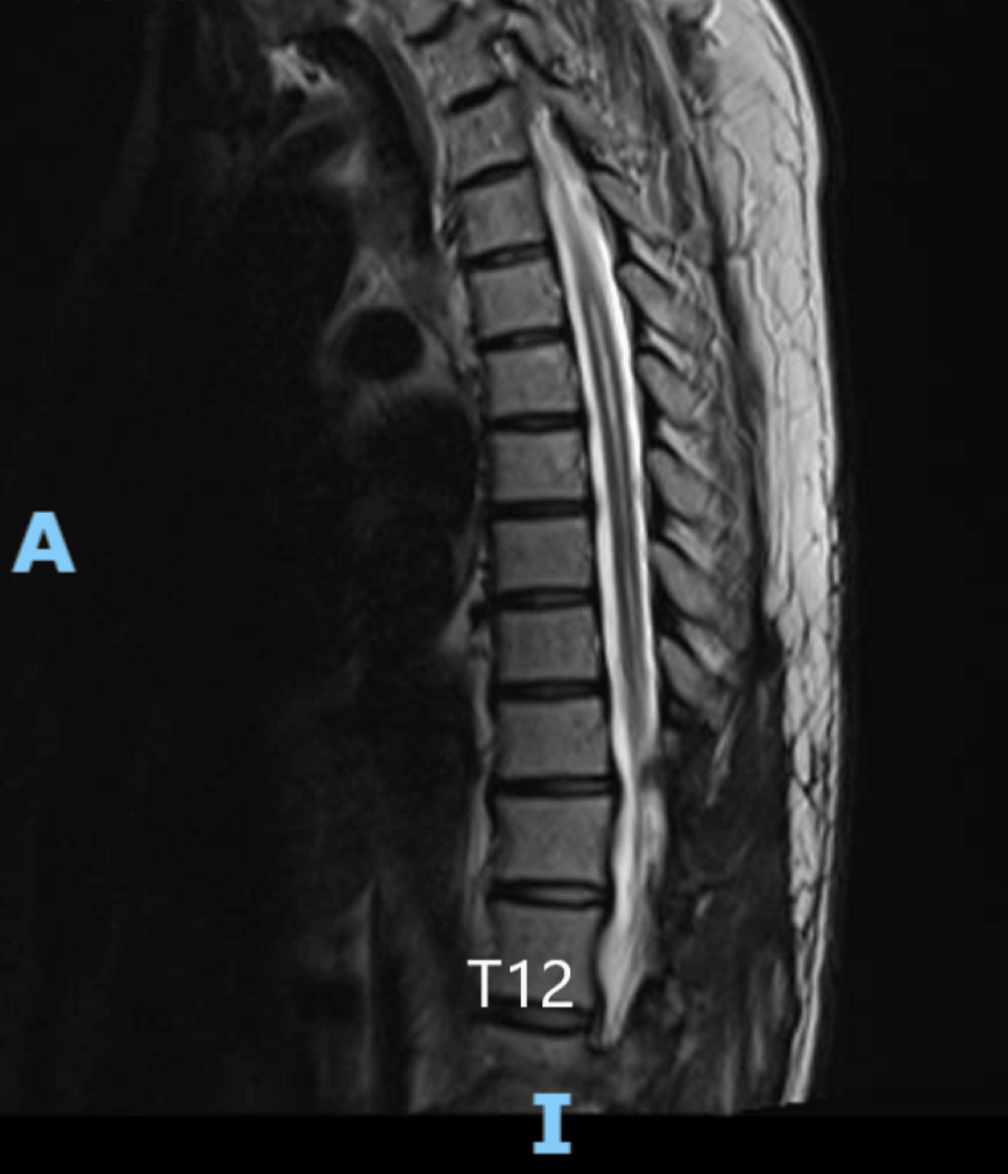
Post-operative sagittal MRI image of intradural lesion at the level of T12 at seven months post operation showing significant improvement of syrinx.

Post-operative MRI (see Figures 12, 13 for post-operative MRIs) revealed a significant reduction in tumour size and extent of syrinx. Similarly, there was a notable absence of metal artefacts despite the use of Synthes plates and screws. She made an uneventful recovery and was discharged on post-operative day seven to rehabilitation. Repeat MRI performed at seven months post operation showed significant improvement of the syrinx (see Figure 14 for post-operative MRI).

## Discussion

Literature review

The existing literature surrounding lamina reimplantation post laminectomy for the treatment of intradural tumours is limited. An initial keyword search with “lamina reimplantation for intradural tumours” revealed several relevant studies (see Table 2). We review the benefits described in these studies.

**Table 2:** Summary of existing literature surrounding lamina reimplantation post laminectomy for the treatment of intradural tumours. ROM: range of motion; VAS: visual analogue scale.

Author	Title	Type of study	Sample size	Benefits described
Leng et al. (2024) [11]	Combined replantation of the posterior arch of the atlas and bilateral axial lamina in the treatment of intradural schwannoma: a case report	Case report	1	Preservation of the mobility of the atlantoaxial segment, avoidance of the development of postoperative fibrosis
Liu et al. (2023) [10]	Comparison of the clinical effects of lamina replantation and screw fixation after laminectomy in the treatment of intraspinal tumours	Case series	58	Reduction in operation time, occurrence of postoperative cerebrospinal fluid leakage, iatrogenic spinal stenosis, posterior soft tissue adhesion and adjacent segment degeneration (ASD)
Jiang et al. (2023) [12]	Clinical and biomechanical study of laminoplasty for thoracic and lumbar intradural tumors	Case series	50	Better maintenance of stability of the spine, preservation of spinal ROM, and reduction in CSF leakage
Dai et al. (2023) [13]	Replantation of lamina spinous process ligament complex and miniature titanium plate shaping internal fixation in the treatment of tumors in the spinal canal	Case series	43	Effective reconstruction of the spinal canal and posterior column structure, reduction of the incidence of cerebrospinal fluid leakage and secondary spinal stenosis
Wang et al. (2021) [14]	Application of open-door laminoplasty with ARCH plate fixation in cervical intraspinal tumors	Case series	38	Lower incidence of spinal deformities and an absence of epidural scar tissue
Duan et al. (2021) [15]	Comparison of total laminectomy and pedicle screw internal fixation with ultrasonic- and microscopic-assisted laminectomy replantation for tumors of the lumbar spinal canal: a retrospective study of 60 cases from a single center	Case series	60	Reduction of intraoperative blood loss, postoperative drainage volume, length of hospital stay, and postoperative VAS pain score
Wang et al. (2020) [16]	Application of laminoplasty combined with ARCH plate in the treatment of lumbar intraspinal tumors	Case series	24	Improvement in spinal stability, compressive resistance, anti-bending, anti-shearing, and anti-rotation abilities
Song et al. (2019) [17]	Efficacy analysis of two surgical treatments for thoracic and lumbar intraspinal tumours	Case series	59	Lower blood loss and volume of drainage, shorter surgical time and hospital stay, avoidance of iatrogenic spinal canal stenosis
Lin et al. (2018) [18]	Lumbar laminotomy and replantation for the treatment of adult spinal epidermoid cyst: A case report	Case report	1	Reduction in the risk of iatrogenic lumbar instability
Zhou et al. (2013) [19]	Application of lamina replantation with ARCH plate fixation in thoracic and lumbar intraspinal tumors	Case series	13	Retention of posterior spinal structures, prevention of postoperative bleeding, scar adhesions, instability, subluxation and kyphosis; uncomplicated access when further surgery is required

The key benefits of lamina reimplantation described by existing literature are the increase in spinal stability, epidural scar adhesions, and cerebrospinal fluid (CSF) leakage.

Literature review suggests a positive correlation between the utilisation of lamina reimplantation post laminectomy and an increase in spinal stability. An increase in longitudinal spinal stress resistance and lateral stability in patients who underwent laminectomy reimplantation was reported by Zhou et al. [19], in comparison to patients who underwent total laminectomy. Wang et al. [16] assessed the radiological extent of bony fusion and compared the Oswestry Disability Index (ODI) scale of patients who underwent spinous process-lamina complex reimplantation with titanium screw and ARCH plate fixation, describing a statistically significant improvement in ODI scale rating and a high proportion of “segmental complete fusion” in their group of patients. While this parameter was not actively studied by us, the authors highlight that post-operative imaging did not suggest any complications of spinal instability in our patients. An important limitation to note from our case series is that this technique is only being considered for spine cases where the spine is considered stable despite laminectomy, and further larger-scale studies will be required to investigate potential benefits of reduction in spinal instability, as opposed to an increase in spinal stability reported in the literature.

The reduction of epidural scar adhesions is also a key benefit of this technique. Liu et al. [10] described a statistically significant reduction in post-operative complications, including postoperative CSF leakage, iatrogenic spinal stenosis, and posterior soft tissue adhesion. In a study comparing conventional laminectomy and laminoplasty with replantation of posterior spinous process-lamina complex, Jiang et al. [12] also reported an absence of spinal adhesions in the group of patients who received laminoplasty with replantation, and the presence of adhesions in the group who received conventional laminectomy. The lack of adhesions observed in our patient upon re-entering the spinal canal for repeat surgery is consistent with the findings in the literature and allowed for an uncomplicated second surgical resection of the intradural tumour.

Lastly, the reduction in incidence of CSF leak is also a recognised benefit of lamina reimplantation. As mentioned, Liu et al. [10] reported a reduction in post-operative complications, including post-operative CSF leakage. Similarly, Jiang et al. [12] also described a statistically significant reduction in the incidence of CSF leakage in a group of patients who received laminoplasty with reimplantation as compared to a group of patients who received conventional laminectomy.

Discussion

We discuss the possible benefits of lamina reimplantation post laminectomy in the treatment of intradural tumours. It mitigates the risk of epidural fibrosis while avoiding significant artefacts on post-operative MRI, which are typically associated with metallic implants.

A major side effect of laminectomy procedures is the potential for epidural fibrosis, leading to scarring and adhesions. While the underlying mechanisms behind epidural fibrosis remain poorly understood, several theories have been proposed. Key and Ford suggested that the damaged intervertebral disc fibre was the major contributing factor to epidural adhesion [20], while LaRocca and Macnab proposed that the sacrospinalis muscle was the major contributing source [21]. The current prevailing view is the three-dimensional fibrosis formation theory first proposed by Songer et al. [22]. They posited that the scar tissue around the dura mater originated from three sources: the remnant sacrospinalis muscle, the fibre ring, and the posterior longitudinal ligament post laminectomy [22]. Replacing the lamina post laminectomy reinstates the mechanical barrier between the dura and the posterior structures, which may aid in preventing fibroblast proliferation into the spinal cord, thereby reducing adhesions. There are multiple clinical benefits of reduced incidence of epidural fibrosis, which can theoretically reduce back pain and stiffness, or even FBSS, and can also allow for easier repeat surgery should it be required. In all cases described above, we note the absence of any reported complications and highlight the notable absence of scarring and adhesions seen during repeat surgery when one patient suffered from a tumour recurrence. While larger-scale studies may be required to determine the benefit of lamina reimplantation in reducing the incidence of adhesions, we note this with concurrence with existing studies such as that of Liu et al., which conserved both laminae and ligamentum flavum in their cohort study [23].

Another possible benefit of lamina reimplantation post laminectomy is the fact that there will be fewer artefacts on follow-up imaging, whereas for spinal instrumentation, this could be obscured due to the presence of metallic implants. In particular for intradural tumours, limiting metal artefacts is key as surveillance imaging is needed to monitor the progress of the patient’s recovery and the adequacy of tumour resection. Conversely, avoiding imaging artefacts allows for better monitoring of potential disease recurrence and may contribute to better patient outcomes. In the above cases described, we noted the distinct lack of metal artefacts on follow-up MRI despite the use of Synthes cranial plates and screws. This mitigates the potential limitation of sub-optimal surveillance imaging and may also allow for wider utilisation of Synthes cranial plates and screws for lamina reimplantation in the surgical resection of intradural tumours.

## Conclusions

Lamina reimplantation post laminectomy is an increasingly reliable alternative to traditional laminectomy in the surgical approach of accessing intradural tumours. While achieving adequate tumour exposure and resection, this technique offers postoperative advantages, most notably a reduced risk of epidural fibrosis and dural adhesions. Furthermore, the minimal artefact on follow-up MRI despite the use of Synthes cranial plates and screws is an evident advantage in long-term follow-up, particularly in individuals with tumour progression or recurrence in whom serial imaging is necessary. Importantly, lamina reimplantation described in this case series is limited to use in patients for spine cases, which are considered stable despite laminectomy, and may not be suitable for all patients. Further comparative studies on a larger scale are needed to substantiate these findings and guide best surgical practices.
